# Nanoparticles Induce Changes of the Electrical Activity of Neuronal Networks on Microelectrode Array Neurochips

**DOI:** 10.1289/ehp.0901661

**Published:** 2010-05-10

**Authors:** Alexandra Gramowski, Juliane Flossdorf, Kunal Bhattacharya, Ludwig Jonas, Margareta Lantow, Qamar Rahman, Dietmar Schiffmann, Dieter G. Weiss, Elke Dopp

**Affiliations:** 1 Institute of Biological Sciences, Cell Biology and Biosystems Technology, University of Rostock, Rostock, Germany; 2 NeuroProof GmbH, Rostock, Germany; 3 Institute of Hygiene and Occupational Medicine, University of Duisburg-Essen, Essen, Germany; 4 Institute of Pathology, Electron Microscopic Center, University of Rostock, Rostock, Germany; 5 Institut für Zelltechnologie IZT e.V., Rostock, Germany; 6 Integral University, Lucknow, India

**Keywords:** carbon black, hematite, nanoparticles, neuronal networks, neurotoxicity, titanium dioxide

## Abstract

**Background:**

Nanomaterials are extensively used in industry and daily life, but little is known about possible health effects. An intensified research regarding toxicity of nanomaterials is urgently needed. Several studies have demonstrated that nanoparticles (NPs; diameter < 100 nm) can be transported to the central nervous system; however, interference of NPs with the electrical activity of neurons has not yet been shown.

**Objectives/methods:**

We investigated the acute electrophysiological effects of carbon black (CB), hematite (Fe_2_O_3_), and titanium dioxide (TiO_2_) NPs in primary murine cortical networks on microelectrode array (MEA) neurochips. Uptake of NPs was studied by transmission electron microscopy (TEM), and intracellular formation of reactive oxygen species (ROS) was studied by flow cytometry.

**Results:**

The multiparametric assessment of electrical activity changes caused by the NPs revealed an NP-specific and concentration-dependent inhibition of the firing patterns. The number of action potentials and the frequency of their patterns (spike and burst rates) showed a significant particle-dependent decrease and significant differences in potency. Further, we detected the uptake of CB, Fe_2_O_3_, and TiO_2_ into glial cells and neurons by TEM. Additionally, 24 hr exposure to TiO_2_ NPs caused intracellular formation of ROS in neuronal and glial cells, whereas exposure to CB and Fe_2_O_3_ NPs up to a concentration of 10 μg/cm^2^ did not induce significant changes in free radical levels.

**Conclusion:**

NPs at low particle concentrations are able to exhibit a neurotoxic effect by disturbing the electrical activity of neuronal networks, but the underlying mechanisms depend on the particle type.

The extensive use and development of nanomaterials demand intensified research efforts regarding toxicity and possible health effects of nanomaterials [e.g., nanoparticles (NPs), nanotubes]. The small size of NPs provides them with special properties, such as high surface-to-volume ratio and high surface charge (Linse et al. 2007). In daily life, NPs can be found almost everywhere (e.g., in cosmetics, dusting powder, paints). Because of their small size, NPs can translocate from the interstitial region of the lung after respiratory exposure and can penetrate through the skin after application in cosmetics, reaching different organs of the body, such as liver, spleen, kidney, and brain, through blood circulation (L’Azou et al. 2008).

The potential translocation of different types of NPs into the central nervous system (CNS) via the olfactory pathway has been reported by Oberdörster and Utell (2002) and Nel et al. (2006; for reviews, see Oberdörster et al. 2004, 2009; Papp et al. 2008). Intranasally instilled anatase titanium dioxide (TiO_2_) NPs in female mice translocated into the CNS and caused a potential lesion in the hippocampus region of the brain (Wang et al. 2008). Nanoscale TiO_2_ particles stimulate brain microglia to produce reactive oxygen species (ROS) through oxidative burst and interfere with mitochondrial energy production *in vitro* (Long et al. 2006). To this point, there is no evidence regarding interference of NPs with electrical activity of neurons. High levels of ROS in the brain have been related to the degeneration of neurons and their mitochondria, leading to the development of Alzheimer’s (Sompol et al. 2008) and Parkinson’s (Smith and Wayne 2007) diseases.

Spontaneously active networks in culture have been proposed as a sensitive and efficient model system to study the neuroactive and toxic properties of chemicals (Gramowski et al. 2006b; Gross et al. 1997; Stett et al. 2003). *In vitro* neuronal networks represent a simplified model to study neural electrophysiology (i.e., the functional output of cellular activity in the CNS). Cultured networks coupled to microelectrode array (MEA) neurochips constitute a valuable tool to investigate changes in the electrophysiological activity of neurons in response to chemical exposure. Neuronal networks respond to transmitters, their blockers, and many other pharmacological substances in a histiotypic manner; responses are substance specific and similar to those observed *in vivo* (Bal-Price et al. 2008; Gramowski et al. 2000, 2004, 2006b; Keefer et al. 2001b; Morefield et al. 2000).

In the present study we investigated the influence of NPs on the electrical activity changes in neuronal networks on MEA neurochips. For this study we used three different kinds of NP: carbon black (CB), TiO_2_, and hematite (Fe_2_O_3_). We exposed primary cortical networks cultured on MEA neurochips to the different NPs in an acute concentration-dependent manner. The action potential patterns of the electrical activity of the neuronal networks were recorded and changes in four activity categories—general activity, burst structure, synchronicity, and oscillatory behavior—were quantified by multiparametric pattern analysis. Additionally, deposition and translocation of NPs in the neuronal network were observed using scanning electron microscopy and transmission electron microscopy (TEM), and acute functional toxicity, cytotoxicity, and radical formation were evaluated.

## Materials and Methods

### NP characteristics and used chemicals

TiO_2_ (diameter < 100 nm) and CB (diameter = 55 nm) particles were provided by Degussa GmbH (Essen, Germany) and by Aditya Birla Nuvo Ltd. (Mumbai, India), respectively. The Fe_2_O_3_ NPs (diameter < 100 nm; catalog no. 544884) were purchased from Sigma-Aldrich GmbH (Munich, Germany). For all experiments, NPs were treated by ultrasound sonification.

The surface area of all the NPs was measured using the Brunauer–Emmett–Teller (BET) technique at the Nanolab of Focas Institute (Dublin Institute of Technology, Dublin, Ireland). For BET, the samples were analyzed in a dry state by nitrogen adsorption after degassing at 300°C (Gemini 2360 analyzer; Micromeritics Instrument Corp., Norcross, GA, USA). Zeta potential values were obtained using the Smoluchowski model for analysis, and particle size distribution was measured using the dynamic light-scattering technique (Nano Zetasizer ZS; Malvern Instruments, Worcestershire, UK) after suspending the NPs in fetal bovine serum–free cell culture medium by ultrasonication in an ultrasonic water bath.

### Primary frontal cortex cell culture

Frontal cortex tissue was harvested from embryonic day 15 crl:NMRI mice. After ethyl ether anesthesia, mice were sacrificed by cervical dislocation according to the German Animal Protection Act (1998). Animals were treated humanely and with regard for alleviation of suffering. Frontal cortex tissue was cultured according to the method of Ransom et al. (1977) with minor modifications: DNase I (8,000 U/mL) and papain (10 U/mL) were used for tissue dissociation (Huettner and Baughman 1986). This method has been described in detail previously (Gramowski et al. 2006a).

Primary frontal cortex networks cultured on MEA neurochips routinely develop spontaneous electrical activity, which starts after approximately 3–4 days *in vitro* (Gramowski et al. 2004) in the form of random spiking. This stabilizes after 4 weeks in culture into a synchronized activity pattern composed of a coordinated burst pattern and interburst spiking [see Supplemental Material, Figure 1 (doi:10.1289/ehp.0901661)]. Such networks can remain spontaneously active and pharmacologically responsive for > 6 months and show high interculture repeatability (Gramowski et al. 2006b; Gross 1994).

### MEA neurochips recording

MEA neurochips were provided by the Center for Network Neuroscience (University of North Texas, Denton, TX, USA). These 5 × 5 cm^2^ glass chips have a central 1-mm^2^ recording matrix with 64 passive gold electrodes and indium tin oxide conductors. Fabrication techniques and culture methods have been described previously (Gross 1994; Gross et al. 1985).

The particle concentration range for the three NPs investigated in the electrophysiological studies was chosen based on the effective range on the frontal cortex activity. In preliminary experiments, we determined the complete acute (12 hr) effective range for each NP and determined the accumulating concentrations to optimally cover the effective range. Before application to the MEA neurochip chamber or 12-well chamber, the NPs were dispersed by ultrasound sonification and vortexed.

### Multichannel recording and data analysis

For extracellular recording, MEA neurochips were placed into sterilized constant-bath recording chambers (Gross 1994) and maintained at 37°C. Recordings were made in Dulbecco’s modified Eagle’s medium/10% horse serum at 1 mL chamber volume. The pH was maintained at 7.4 with a continuous stream of filtered, humidified air with 10% CO_2_. Sets of 32 preamplifiers were positioned to either side of the recording chamber, and recording was performed with the multichannel acquisition processor system and a computer-controlled 64-channel amplifier system (Plexon, Inc., Dallas, TX, USA). The total system gain used was 10 K, with a simultaneous 40-kHz sampling rate. Spike identification and separation were accomplished with a template-matching algorithm in real time [see Supplemental Material, Figure 1B,C (doi:10.1289/ehp.0901661)].

High content analysis of the network activity patterns provides a multiparametric description characterizing the changes in four categories: general activity, burst structure, synchronicity, and oscillatory behavior. We quantified the substance-specific activity changes by extracting a total of 35 activity-describing spike train parameters as previously described (Gramowski et al. 2006b). Four of these parameters quantify the concentration–response kinetics in their course, number of phases, slope (Hill coefficient), and 10%, 50%, and 90% effective concentrations (EC_10_, EC_50_, and EC_90_, respectively) of the maximum inhibitory effect induced by the NPs for the frontal cortex spike rate (SR). The remaining 31 parameters [listed in Supplemental Material, Table 1 (doi:10.1289/ehp.0901661)] include parameters representing general activity [e.g., SR and burst rate (BR)] and burst structure (e.g., burst duration and number of spikes within a burst).

For direct comparability, all parameters were normalized for each experiment and each experimental treatment with respect to the corresponding values of the native reference activity. Values were derived from 60-sec bin data from 30 min after stabilization of activity. From one network, between 24 and 101 separate neurons were simultaneously reordered.

The changing BR as a function of the concentration was fitted to a sigmoidal dose–response curve in order to obtain dose–response curves for each experiment. Where the activity changes showed a biphasic or multiphasic behavior in the dose–response curve, the phases were fitted and quantified separately.

### Raster scanning electron microscopy (REM)

Neuronal networks on MEA neurochips were incubated for 24 hr with the three NP types, fixed in 4% glutaraldehyde for 1 hr, washed with phosphate-buffered saline (PBS), postfixed with 1% osmium tetroxide (OsO_4_), dehydrated in a graded series of acetone, and dried with a critical point dryer. The sample was coated with gold in a sputter coater and studied in the REM (DSM 960A; Carl Zeiss AG, Oberkochen, Germany).

### TEM

Before application of NPs to the cortical networks on MEA neurochips, the NPs were dispersed by ultrasound sonification and vortexed. For incubation, the networks were washed twice with serum-free medium and incubated for 24 hr with the three types of NPs (CB, 10 μg/cm^2^; TiO_2_, 10 μg/cm^2^; Fe_2_O_3_, 5 μg/cm^2^). At the end of incubation, the monolayers were washed with serum-free medium and fixed with 4% glutaraldehyde in 0.1 M sodium phosphate buffer (pH 7.4) for 1 hr at 4°C. The fixed monolayers were removed from the culture dishes with a rubber policeman, washed in 0.1 sodium phosphate buffer, and postfixed with 1% OsO_4_ in the same buffer for 1 hr at 4°C. The sample pellets were then washed in PBS and dehydrated. After postfixation, the samples were washed in PBS, dehydrated in a graded series of acetone, and embedded in the epoxy resin Araldite (Fluka, Buchs, Switzerland). Ultrathin sections were cut with an Ultracut S ultramicrotome (Leica, Wetzlar, Germany), mounted on copper grids, stained with uranyl acetate and lead citrate, and studied in an EM 902 A TEM with electron energy loss spectroscopy (EELS) or Libra 120 TEM with EELS and electron-dispersive X-ray spectroscopy (Carl Zeiss AG). Digital pictures were taken with a 2K CCD camera (Proscan, Lagerlechfeld, Germany). The particle size was measured using the EFTEM software package (Zeiss-SIS, Jena, Germany) at a magnification of 15,000×.

### Measurement of ROS

ROS production was detected using the dihydrorhodamine 123 (DHR), which is oxidized by hydrogen peroxide (H_2_O_2_), hypochlorous acid (HClO^•^), and peroxynitrite anion (ONOO^•^) to the fluorescent dye rhodamine 123. We measured the fluorescence intensity of reduced rhodamine 123 at 525-nm emission wavelength by a flow cytometer (EPICS XL-MCL4; Beckman Coulter, Krefeld, Germany) as previously described by Lantow et al. (2006). After exposure to the different NPs at concentrations ranging from 0.5 to 10 μg/cm^2^ for 24 hr, neuronal cultures were incubated with 1 μM DHR (end concentration) in Hank’s balanced salt solution (0.9% NaCl, 14 mM HEPES, pH 7.4) for 25 min at 37°C in the dark. ROS production was immediately measured by flow cytometry. For data analysis, we used EXPO32 MultiCOMP analysis software (version 1.2; Beckman Coulter). Results are expressed as mean ± SD from three independent experiments, in which all measurements were carried out in triplicate.

### Statistical analysis

The electrophysiological results are expressed as series means ± SE. The absolute parameters’ distributions were tested for normality. For the electrophysiological studies, we assessed the level of significance after compound application using SPSS statistical software, version 17.0 (SPSS, Chicago, IL, USA). Significant changes induced by substance application were tested by analysis of variance (ANOVA) followed by Dunnett’s multiple comparison post hoc test with native activity as the common control.

To determine the EC_50_, standard logistic concentration–response curves (either one or the sum of two, depending on the data) were fitted to the data points using the nonlinear regression algorithm of the Solver module in Microsoft Excel (Microsoft Corporation, Redmond, WA, USA). The end point was set to the maximum effect of the SR and BR network changes induced by the respective NP.

For the ROS measurements, we determined statistical differences between the means using the ANOVA test. Values of *p* < 0.05 were considered significantly different from control.

## Results

### Physicochemical analysis

Table 1 shows the surface area, zeta potential, and average hydrodynamic diameter (particle size distribution) of the NPs used. Electron-dispersive X-ray analysis for detecting the elemental composition of the NPs revealed a carbon peak for CB NPs, an iron peak for Fe_2_O_3_ NPs, and a titanium peak for TiO_2_ NPs (Table 1). Analysis of REM images of dry NPs are shown in Supplemental Material, Figure 2 (doi:10.1289/ehp.0901661).

### Influence of CB NPs on electrical activity of cortical networks

**Figure 1:**
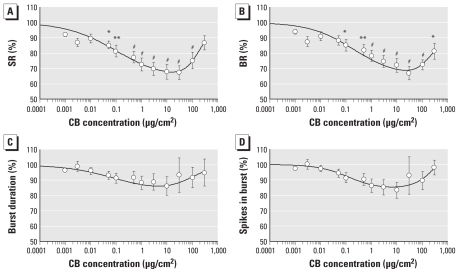
Concentration-dependent changes in four general activity parameters of neuronal networks after exposure to CB NPs. (*A*) SR. (*B*) BR. (*C*) Burst duration. (*D*) Spikes in burst. *p < 0.05. **p < 0.01. ^#^*p* < 0.001.

We tested CB in a concentration range of 0.001–300 μg/cm^2^ (*n* = 8). The high interculture repeatability of the electrophysiological activity response induced by the three NPs is demonstrated in Supplemental Material, Figure 3 (doi:10.1289/ehp.0901661). CB induced biphasic concentration-dependent activity changes in the electrical activity of cortical networks. At low concentrations (0.03–100 μg/cm^2^), CB evoked a reduction of the general activity (phase 1). This initial activity drop was followed by an increase of the cortical network SR activity at higher CB concentrations (10–300 μg/cm^2^) (phase 2; Figure 1). In phase 1 the significant decrease in the general activity started at 0.03 μg/cm^2^ SR, 92.0 ± 1.3%; BR, 93.9 ± 1.4%). We observed a maximum decrease in the general activity at 30 μg/cm^2^ (SR, 67.2 ± 4.3%; BR, 66.1 ± 4.4%). To determine the EC_50_, we fitted the dose–response curves to the maximum inducible effect of the NPs. The initial value was set to 100% and the final value was not fixed. Fitted EC_10_, EC_50_, and EC_90_ values for SR in this activity decline (phase 1) were 0.001, 0.915, and 851 μg/cm^2^, respectively (Table 2). The Hill coefficient for the dose–response curve was 0.32 for the phase 1 slope. During phase 1, activity changes decreased with rising CB concentrations beginning at 10 μg/cm^2^, which is reflected by an increasing coefficient of variation (CV) averaged over the network (CV_net_) for the BR (174.0 ± 24.1%; see Supplemental Material, Figures 4 and 5). These changes were accompanied by a decomposition of the network oscillation at 20 μg/cm^2^ and upward, which is reflected by the increasing CV averaged over time (CV_time_) for the BR (203.0 ± 26.3%). The number of bursting units remained unaltered.

The activity decrease of cortical networks was followed by a CB-induced switch in activity changes characterized by an enhancement of activity at higher concentrations (phase 2). This phase 2 activity induction occurred at 100–300 μg/cm^2^ CB (Figure 1, Table 2). SR and BR showed a maximum increase of 16.3% and 13.1%, respectively. These activity changes were not accompanied by changes in the burst structure. Within this activity enhancement, network synchronization and network oscillation behavior became more coordinated. The number of bursting units remained unaltered. Of the 31 activity parameters, 16 showed significant changes [see Supplemental Material, Figures 4 and 5 (doi:10.1289/ehp.0901661)].

### Influence of CB NPs on cortical network morphology

**Figure 2:**
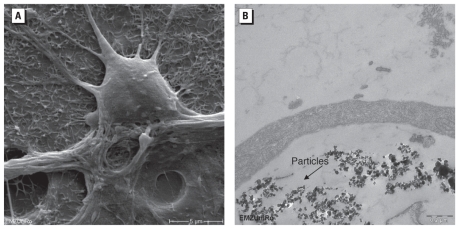
REM (*A*) and TEM (*B*) images of neuronal network cells exposed to 10 μg/cm^2^ CB NPs for 24 hr. (*A*) The typical network appearance with a glial cell carpet and neurons on top. (*B*) CB particles were bound to the cell surface and accumulated inside the cells. In *A*, bar = 5 μm; in *B*, bar = 0.2 μm.

Studies of ultrathin sections of CB-treated neuronal networks in TEM demonstrated the presence of the NPs in the cells (Figure 2). A few CB particles with a diameter of about 55 nm were bound to the cell surface. We detected no obvious cell damage or injury after 24-hr exposure.

### Influence of CB NPs on intracellular ROS formation

**Figure 3:**
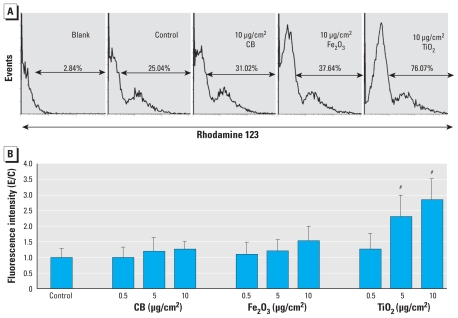
ROS levels after exposure of neuronal networks to different concentrations of CB, Fe_2_O_3_, or TiO_2_ NPs for 24 hr. (*A*) Cell autofluorescence (“Blank”) and the changes in fluorescence intensity and peak distribution of rhodamine 123 analyzed by flow cytometry (DHR assay), in control cells and in cells exposed to NPs. (*B*) ROS formation in neuronal cells after exposure to different concentrations (0.5–10 μg/cm^2^) of NPs. Values shown are the mean ± SD of experiment/control (E/C) data from three independent experiments (and preparations) performed in triplicate. ^#^*p* < 0.001.

We investigated the effects of the different NPs at concentrations of 0.5, 5, and 10 μg/cm^2^ after 24-hr exposure on the formation of ROS (Figure 3). Results show that exposure to CB NPs did not induce changes in the ROS level in neuronal networks.

### Influence of Fe_2_O_3_ NPs on electrical activity of cortical networks

**Figure 4:**
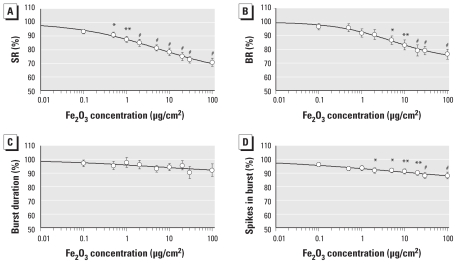
Changes in four general activity parameters of neuronal networks after exposure to Fe_2_O_3_ NPs. (*A*) SR. (*B*) BR. (*C*) Burst duration. (*D*) Spikes in burst. *p < 0.05. **p < 0.01. ^#^*p* < 0.001.

We tested Fe_2_O_3_ NPs for neurotoxic potential in concentrations of 0.1–100 μg/cm^2^ (*n* = 16 per treatment). These NPs caused less pronounced changes in the cortical activity network patterns than did CB NPs. The general activity showed a steady reduction with rising Fe_2_O_3_ concentrations, with a maximum decline in SR and BR to 71.0 ± 3.1%, and 76 ± 3.7%, respectively, at 100 μg/cm^2^ (the highest concentration tested) (Figure 4). At a concentration of 0.5 μg/cm^2^, Fe_2_O_3_ induced a significant decrease in activity. We calculated the EC_10_, EC_50_, and EC_90_ values for the SR as 0.025, 6.6, and 1,760 μg/cm^2^, respectively (Table 2). This effect on SR was accompanied by changes in the burst structure (specifically, a decrease in the number of spikes in burst to 88.1 ± 2.4%) (Figure 4) and by a reduction in synchronicity and oscillatory behavior [see Supplemental Material, Figures 6 and 7 (doi:10.1289/ehp.0901661)]. However, even at the highest concentration tested, all neurons in the network remained actively bursting. In 16 of the 31 activity parameters, Fe_2_O_3_ induced significant changes.

### Influence of Fe_2_O_3_ NPs on cortical network morphology

**Figure 5:**
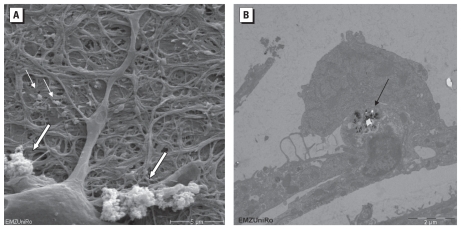
REM (*A*) and TEM (*B*) images of neuronal network cultures exposed to 5 μg/cm^2^ Fe_2_O_3_ NPs for 24 hr. In *A*, particles were detected as agglomerates (large arrows) and as single particles (small arrows); bar = 5 μm. In *B*, NPs were detected inside cells (arrow); bar = 2 μm.

Binding of Fe_2_O_3_ to the cell surface of neuronal network cells and particle uptake by endocytosis was visible by electron microscopy (Figure 5). In about 1% of investigated cell preparations, we found NPs in what looked like a loose neuropil also containing synapses (Figure 5B). A differentiation of glial cells or the special subtypes of neurons was not possible in TEM.

### Influence of Fe_2_O_3_ NPs on intracellular ROS formation

Similar to results with CB, exposure of neuronal networks to Fe_2_O_3_ NPs did not induce a statistically significant increase of ROS formation up to a tested concentration of 10 μg/cm^2^ and an exposure time of 24 hr (Figure 3).

### Influence of TiO_2_ NPs on electrical activity of cortical networks

**Figure 6:**
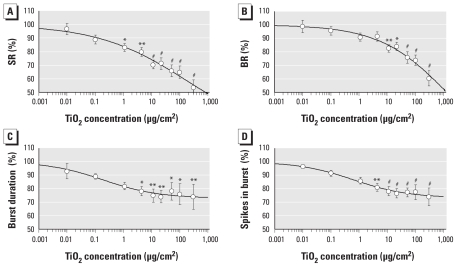
Changes in four general activity parameters of neuronal networks after exposure to TiO_2_ NPs. (*A*) SR. (*B*) BR. (*C*) Burst duration. (*D*) Spikes in burst. *p < 0.05. **p < 0.01. ^#^*p* < 0.001.

We tested TiO_2_ NPs at concentrations ranging from 0.01 to 300 μg/cm^2^ (*n* = 10). TiO_2_ NPs caused severe inhibition of the general electrical network activity, with 1 μg/cm^2^ TiO_2_ NPs. At 300 μg/cm^2^, SR and BR dropped to 53.3 ± 5.5%, and 60.3 ± 5.7%, respectively (Figure 6). The EC_10_, EC_50_, and EC_90_ values for the SR in this activity decrease were 0.11, 665, and 3,840 μg/cm^2^, respectively (Table 2). With rising TiO_2_ concentrations, the synchronicity and the oscillatory regularity of the network activity pattern were reduced. This was observed at 300 μg/cm^2^ in the increasing BR CV_time_ (216.9 ± 27.9%) and CV_net_ (231.6 ± 40.1%) [see Supplemental Material, Figures 8 and 9 (doi:10.1289/ehp.0901661)]. In addition, the burst structure changed with increasing concentrations. The burst duration was markedly reduced to 73.9 ± 9.2% and the number of bursts to 74.0 ± 6.5%. However, all neurons remained bursting over the time of the concentration–response experiments. In 17 of the 31 activity parameters, TiO_2_ induced significant changes.

### Influence of TiO_2_ NPs on cortical network morphology

**Figure 7:**
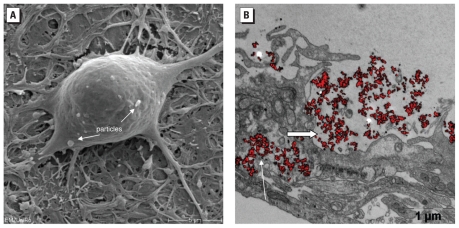
REM (*A*) and TEM (*B*) images of neuronal networks exposed to TiO_2_-NPs. (*A*) Neuronal cell with internalized TiO_2_ NPs (arrows). (*B*) Accumulation of particles inside lysosomes (large arrow) and residual bodies (small arrow). In *A*, bar = 5 μm; in *B*, bar = 1 μm.

Figure 7 demonstrates the uptake of TiO_2_ into neuronal network cells as well as an accumulation of particles or particle agglomerates in a space near the cell surface.

### Influence of TiO_2_ NPs on intracellular ROS formation

TiO_2_ NPs induced a statistically significant and concentration-dependent increase in ROS formation in neuronal cells compared with the untreated control. They induced a substantial increase of ROS-producing cells (76.07%) compared with CB (31.02%) and Fe_2_O_3_ (37.64%) NPs (Figure 3A) and considerably higher ROS levels than did Fe_2_O_3_ NPs at the same concentration (Figure 3B).

## Discussion

Properly anticipating the potential adverse effects caused by NPs requires a fundamental understanding of their physical properties and distribution, as well as a detailed multiparametric analysis of their cellular effects.

### Particle properties

The dynamic light-scattering analysis demonstrated that NPs suspended in serum-free liquid medium formed agglomerates; because of that, the hydrodynamic diameter increased. This can be due to the presence of weak van der Waals forces or strong chemical bonds between the individual NPs. Agglomeration of suspended NPs depends on their zeta potential, which in turn relies on several factors such as ionic strength, pH, surface charge, and surface coating (Jiang et al. 2009). Stable dispersions of NPs in solution occur only at zeta potentials greater than 30 mV (positive or negative) (Wagner et al. 2007). In the present study, we observed a clear correlation between zeta potential and agglomeration state of the NPs by determining the hydrodynamic diameter of the NPs. CB NPs with a low zeta potential of 14 mV (positive or negative) indeed showed a high degree of agglomeration, whereas the other types with zeta potentials of 29 mV and 49 mV (positive or negative) remained monodisperse to a large extent.

### Effects on the electrical network activity *in vitro*

For the activity changes in frontal cortex networks, SR and BR were the predominant parameters quantifying the specific concentration-dependent responses. The results indicate that even very low particle concentrations (1–10 ng/cm^2^) can disturb the electrical activity of neuronal networks *in vitro*. All three types of NPs induced significant changes of SRs and BRs, whereby CB had the highest efficiency at the lowest particle concentrations, followed by Fe_2_O_3_ and TiO_2_ NPs. In contrast, TiO_2_ induced the strongest inhibition in the network activity. CB NPs induced a significant activity drop at lower concentrations (1–100 μg/cm^2^), whereas higher concentrations (100–300 μg/cm^2^) induced an increase in the general activity, synchronicity, and oscillatory regularity. Although concentrations > 100 μg/cm^2^ will rarely occur, this condition would resemble the electrical activity pattern of epileptic seizures (Keefer et al. 2001a). In the present study we did not observe this biphasic pattern for TiO_2_ and Fe_2_O_3_ NPs.

### Particle concentrations

Although *in vivo* to *in vitro* comparisons are difficult in general, our electrophysiological *in vitro* findings correlate to some degree with *in vivo* studies. Exposure concentrations used for CB and TiO_2_ studies are in the range of 6–10 mg/m^3^ (Gallagher et al. 1994), which corresponds to 6–10 ng/cm^2^ in our *in vitro* experiments. The recommended exposure limit suggested by the U.S. National Institute for Occupational Safety and Health (1988) is 3.5 mg/m^3^ for CB and < 5 mg/m^3^ (corresponding to 5 ng/cm^3^) for personal inhalable dust exposure. The lowest effective CB concentrations in the present study were in the range of a few nanograms per square centimeter. However, concentrations of NPs or metals that reach the brain are much lower than exposure concentrations. For example, in mice exposed intranasally to 500 μg TiO_2_ NPs (rutile, 80 nm/animal; anatase, 155 nm/animal) for 30 days, Wang et al. (2008) found 280 ng TiO_2_ NPs per gram of brain tissue. Several studies regarding size-dependent accumulation and distribution were carried out with gold NPs. Sonavane et al. (2008) concluded from their studies that gold NPs < 50 nm are able to cross the blood–brain barrier (BBB). The TiO_2_ NPs used by Wang et al. (2008) were > 50 nm and were found in the brains of exposed mice. Terentyuk et al. (2009) found 0.6 μg/mL gold NPs (15 nm) in rat brain after intravenous injection of 57 μg/mL gold NPs. Smaller NPs cross the BBB more easily than do larger NPs. The NPs used in our own experiments were relatively large (50–100 nm); thus, it might be that the effects we observed would be even stronger using NPs < 50 nm. Also, NPs seem to bioaccumulate in inner organs (including brain) after exposure. In our study, we observed effects after short-term exposure, but long-term exposure might even cause stronger effects.

The concentrations at which neuronal networks respond to neuropharmaceuticals *in vitro* correlate well with concentrations known to be active *in vivo* or measured in treated patients (Keefer et al. 2001b). This has also been shown for the antifouling agent trimethyltin chloride (Gramowski et al. 2000); for anandamide, the endogenous ligand of the CNS cannabinoid receptors (Morefield et al. 2000); and for pyrethroid pesticides (Meyer et al. 2008). Therefore, networks of neurons on MEA neurochips can be considered to represent robust, fault-tolerant, spontaneously active dynamic systems with high sensitivity to their chemical environment (Gross et al. 1997) and have a predictive value that may be useful for regulatory testing (Bal-Price et al. 2008).

### BBB and entry into the CNS

The *in vitro* MEA neurochip model system lacks the BBB, and it does not take into consideration the bioavailability of compounds. However, we conducted the measurements in this study under serum media conditions. Therefore, the potential adsorption of NPs to plasma proteins needs to be considered (Wasdo et al. 2008), which means that the freely available particle concentration may be even lower. As mentioned above, the olfactory nerve pathway can be considered another critical portal of NPs entry to the CNS, especially under conditions of elevated environmental or occupational exposure (Fechter et al. 2002; Gianutsos et al. 1997; Henriksson and Tjälve 2000).

### Transport and distribution in the CNS

The high endocytic activity of nerve cells at synapses makes the CNS prone to increased NP incorporation, distribution, and subsequent interference in neuronal functions. Axoplasmic transport provides the mechanism to bidirectionally translocate not only organelles and viruses but also injected NPs, which are widely used neuroanatomists to trace nerve fiber connectivity (Weiss and Gorio 1982). Oberdörster et al. (2009) summarized the possible translocation pathways of NPs and evaluated the hazardous potential of NPs. De Lorenzo (1970) demonstrated that intranasally instilled silver-coated colloidal gold particles (50 nm) translocate anterogradely in the axons of the olfactory nerves to the olfactory bulbs. The NPs even crossed synapses in the olfactory glomeruli and reached mitral cell dendrites within 1 hr after intranasal instillation. Their velocity was 2.5 mm/hr, and they were preferentially located in mitochondria, raising a major concern regarding their toxicity (Oberdörster et al. 2004, 2005). Yu et al. (2007) showed that inhaled 20-nm nanogold particles accumulate in the olfactory bulb of rats. After 15 days, significant accumulations of gold particles were detected in the septum and entorhinal cortex, both brain structures receiving direct neuronal projections from the olfactory bulb and important in attention and new memory formation. Particles with diameters > 1 μm do not enter the olfactory nerve (Oberdörster et al. 2005).

### Intracellular localization

In the present study, our REM data show NPs bound to the cell surface, and the TEM results indicate that NPs are taken up into neuronal network cells. These data are only qualitative and preliminary, so more detailed studies are needed to clarify how the NPs enter the cells, what the neuron-to-glia ratio is for NP entry, and whether only internalized NPs or also those attached to the neuronal or axonal membranes influence the action potential propagation and general electrical activity.

### Significance of the zeta potential

The surface charge is one of the major physical properties of the NPs and can be measured only indirectly through the zeta potential, which is a function of the surface charge of the particle or any adsorbed layer at the interface and the nature and composition of the surrounding medium in which the particle is suspended. In our study using NPs suspended in serum-free media, we found a high positive zeta potential for TiO_2_ NPs compared with the negative zeta potential of Fe_2_O_3_ and CB NPs. The activity drop of the neuronal networks at low particle concentrations was most pronounced for CB, followed by Fe_2_O_3_ and TiO_2_ NPs.

Using engineered NPs with different surface charges, Lockman et al. (2004) and Kreuter (2004) studied the influence of the zeta potential and the applied NP concentration on BBB integrity and evaluated BBB integrity and NP brain permeability by *in situ* rat brain perfusion. Neutral NPs and low concentrations of anionic NPs had no effect on BBB integrity, whereas high concentrations of anionic NPs and low concentrations of cationic ones disrupted the BBB function, indicating the importance to consider particle charges.

### Role of ROS

For ROS production in the present study, the different NP species exhibited a potency sequence opposite that for disturbing electrical activity. TiO_2_ was most effective, followed by Fe_2_O_3_ and CB. Therefore, ROS production cannot explain the functional neurotoxicity (e.g., electrophysiological changes) that we observed. Results for ROS production in this study are in good agreement with previous investigations in which significant differences were reported in intracellular radical formation in human lung cells exposed to TiO_2_ and Fe_2_O_3_ NPs (Bhattacharya et al. 2009). Although TiO_2_ NPs induced a concentration-dependent increase in ROS formation, the effect was delayed in Fe_2_O_3_-exposed BEAS-2B cells and was significant only for the particle concentration of 5 μg/cm^2^ (Bhattacharya et al. 2009). Interestingly, in the present study we found that CB NPs did not increase ROS levels in neuronal networks after 24 hr exposure. Similar observations were reported by Yang et al. (2009), who compared different nanomaterials and reported a low potential for ROS formation in CB NPs.

## Conclusion

Using co-cultures of primary neurons and glial cells, which form stable and electrically active neuronal networks on MEA neurochips, we found that electrophysiological studies are suitable to measure changes caused by NP exposure. NPs are able to induce acute functional neurotoxicity at low particle concentrations, whereas formation of intracellular free radicals may require higher particle concentrations and longer exposure times. To the best of our knowledge, this is the first study demonstrating acute functional neurotoxic effects of NPs. The results justify further studies with MEA neurochips to analyze the underlying mechanism of action and the long-term consequences of low-dose NP exposure.

## Figures and Tables

**Table 1:** Chemical and physical properties of the NPs used.

NP type	Elements	Weight (%)	Diameter (REM, nm)	Average hydrodynamic diameter (nm)	Zeta potential (mV)	Surface area (m^2^/g)
CB	C	88.6	55	575.2	−14.5 mV	123.0 ± 0.01
	O	10.8				
	S	0.65				
Fe_2_O_3_	Fe	78.7	< 100	50	−28.68 mV	34.39 ± 0.17
	O	21.3				
TiO_2_	Ti	56	< 100	91	+48.8 mV	49.71 ± 0.19
	O	41				
	C	3				

S, sulfur.

**Table 2:** Dose–response curve parameters of SR alterations by NPs.

Substance	EC_10_	EC_50_	EC_90_	Hill coefficient
CB				
Phase 1	0.001	0.915	851	0.321
Phase 2	9.10	206	4,660	0.704
Fe_2_O_3_	0.025	6.62	1,760	0.393
TiO_2_	0.115	665	3,840	0.254

EC_10_, EC_50_, and EC_90_ values (μg/cm^2^) and Hill coefficients (slopes) are given for acute exposure with CB (*n* = 8), Fe_2_O (*n* = 16), and TiO_2_ (*n* = 10) NPs.
